# Gender and the Publication Output of Graduate Students: A Case Study

**DOI:** 10.1371/journal.pone.0145146

**Published:** 2016-01-13

**Authors:** Michele Pezzoni, Jacques Mairesse, Paula Stephan, Julia Lane

**Affiliations:** 1 GREDEG, University of Nice Sophia Antipolis, Nice, France; 2 CRIOS, Bocconi University, Milan, Italy; 3 BRICK, Collegio Carlo Alberto, Torino, Italy; 4 CREST-ENSAE, Paris, France; UNU-MERIT, Maastricht University, Netherlands; 5 NBER, Cambridge, Massachusetts, United States of America; 6 Andrew Young School, Georgia State University, Atlanta, Georgia, United States of America; 7 New York University, New York, New York, United States of America; 8 University of Strasbourg, Strasbourg, France; 9 University of Melbourne, Melbourne, Australia; Katholieke Universiteit Leuven, BELGIUM

## Abstract

We examine gender differences among the six PhD student cohorts 2004–2009 at the California Institute of Technology using a new dataset that includes information on trainees and their advisors and enables us to construct detailed measures of teams at the advisor level. We focus on the relationship between graduate student publications and: (1) their gender; (2) the gender of the advisor, (3) the gender pairing between the advisor and the student and (4) the gender composition of the team. We find that female graduate students co-author on average 8.5% fewer papers than men; that students writing with female advisors publish 7.7% more. Of particular note is that gender pairing matters: male students working with female advisors publish 10.0% more than male students working with male advisors; women students working with male advisors publish 8.5% less. There is no difference between the publishing patterns of male students working with male advisors and female students working with female advisors. The results persist and are magnified when we focus on the quality of the published articles, as measured by average Impact Factor, instead of number of articles. We find no evidence that the number of publications relates to the gender composition of the team. Although the gender effects are reasonably modest, past research on processes of positive feedback and cumulative advantage suggest that the difference will grow, not shrink, over the careers of these recent cohorts.

## Introduction

Despite growing participation of women in science, gender differences in publications, an important measure of scholarly productivity, persist[1,2]. Yet little is known regarding the extent to which gender differences are observed in graduate school, or if differences only begin to emerge over the course of the career. Likewise, little is known as to whether the gender of the advisor and the gender pairing between the advisor and graduate student play a role in the scholarly productivity of the student. Nor do we know whether the gender composition of the team that the student works with while training is correlated with publishing productivity.

In this paper we examine differences in publications among the six PhD student cohorts 2004–2009 at the California Institute of Technology (Caltech), a highly selective research intensive university, using a new dataset that includes longitudinal information, such as occupation, on all individuals paid on federal grants and connects them with their publications. We focus on graduate students given the key role that publications at the time of training play in subsequent placement outcomes and career trajectories. We are particularly interested in how the publications of graduate students relate to their gender and the gender of the advisor. We are also interested in whether the gender effects of the advisor are mediated by the gender of the graduate student.

The data enable us to go one step further in studying the publishing productivity of graduate students by constructing a detailed measure of teams at the advisor level. We use this measure to explore how the gender composition of the team relates to publications. We see our work as an important contribution given the key role that teams play in affecting productivity, be it that of scientists[3–5], soccer players [6], supermarket checkers [7], or fruit pickers [8]. The exploration of teams is particularly relevant given the important role that graduate students and postdoctoral fellows play in teams in science [9,10] and work that relates team performance to the gender composition of the team[11–13].

## Framing

Numerous studies have examined gender differences in the productivity of scientists [1,14–17]. Virtually none, however, have studied whether at the earliest stage of a scientist’s professional career—during graduate school—there is a gender difference in publication output, and if differences exist, the effect of systematic gender variation in graduate students’ professional environment on publication outcomes. Even small early career differences can have substantial later career effects, given what is known concerning processes of cumulative advantage and positive feedback mechanisms in science[18–20].

The gender of the advisor may affect mentoring, expectations and the evaluation of trainees’ competencies. A study of US faculty in engineering, the natural sciences and psychology in the early 2000’s found women advisors, for example, to “place significantly more emphasis on giving help to advisees” than male faculty did. The same study found male faculty members to meet more frequently with students and less by design, such as setting specific times for meetings, than did women faculty members [21]. Male faculty were also found to be less likely to see their ideal relationship with a student to be that of mentor-mentee, and more likely to see it as a collegial relationship, than did female faculty members. Male and female faculty at research-intensive institutions in the US rated male student applicants as significantly more competent than female students for a position of lab manager [22]. They also offered more mentoring to male applicants than female applicants. Mentoring, in turn, has been shown to affect subsequent professional outcomes of women in economics[23]. A recent experiment [24], however, found that, regardless of gender, faculty in biology, economics, engineering, and psychology working at a large number of US institutions had a 2:1 preference for hypothetically hiring female applicants over male applicants for a tenure track assistant professor position.

The gender match between advisors and advisees may also be important. Gender, for example, plays a role in how dyads evaluate scientific expertise. A study of 60 teams working in multidisciplinary research centers at a US university found men to give higher ratings to the expertise of other males on their scientific team regardless of the education level of the men. Women were found to give higher ratings to men who are highly educated than they do to women who are highly educated [11]. The gender pairing between faculty and advisee also plays a role in faculty evaluation of factors seen as contributing to success. When evaluating male students, for example, a study in the early 1990s of faculty in doctoral granting departments in science and engineering at US universities found women to put more emphasis on external factors, such as graduating from an elite institution or being aligned with an important faculty member than did male faculty members; no difference was found in terms of how male and female advisors rate the importance of internal factors, such as intelligence and hard work, of male students. For women students, both male and female advisors saw external factors playing a more important role than for male students [25]. Recent work finds gender pairings to be based on gender and accomplishments: males in the US train few women in the biomedical sciences relative to their presence in the training pool: the effect is strongest among elite male faculty[26]. Past research that examines how gender pairing at the graduate level in science and engineering relates to productivity, focuses on the productivity of the advisor, not that of the student, and finds the productivity of male advisors to be an increasing function of the number of male students [16].

The characteristics of the team with which a graduate student works may also affect publication output, since teams perform better when members make the best use of others’ expertise. When deference among members of multidisciplinary teams working at a research university is based on social affinity, (such as gender), the performance of scientific teams suffers while deference based on task contributions enhances performance [27]. Deference based on ethnicity may partly explain why the publication performance of teams, measured by the number of citations or the Impact Factor of the journal in which the publication appeared, is negatively related to the lack of ethnic diversity among the coauthors [28]. Productivity of teams at a US National Laboratory,[29], however, was not found to be related to gender diversity of the team. The gender finding may relate to the mixed contributions that females bring to the team. On the plus side, and as demonstrated by [12] in a study of 40 teams, female dominated teams exhibit higher “collective intelligence,” which, although uncorrelated with the average or maximum intelligence of individual members of the group, explains the performance of the group on a number of tasks. On the negative side, a study found that groups composed exclusively of women underperform groups of other gender configurations in terms of decision making [13]. Study subjects were MBA students and undergraduate students. The performance of individual members of the group may be mediated by their gender and the gender composition of the group. Female freshmen engineering students, for example, exhibit greater participation in female majority groups or in gender parity groups [30]; females randomly assigned to single sex classes in economics at the University of Essex were more likely to pass their first year courses and to score higher on required second year classes than peers who attended coeducational classes [31].

## Materials and Methods

This research and the research design were approved by the Institutional Review Board at the American Institutes of Research. Consent of subjects was not required by the IRB; records of all study subjects were anonymized and de-identified prior to analysis.

We use UMETRICS data to examine the role of gender. UMETRICS data consist of longitudinal information on the researchers directly supported on federal grants, and the vendors who are providing goods and services to support those grants. The data were originally generated as part of the STARMETRICS partnership between 5 federal science agencies, the White House Office of Science and Technology Policy, and over 90 U.S. research universities. The researchers and administrators at the Committee on Institutional Cooperation (CIC) universities have developed an enhanced version of the data so that researchers can link to external datasets and generate better data to model the production and impact of science[32].

The specific dataset is derived from detailed payroll data of Caltech. It captures longitudinal quarterly data on all individuals (and their occupations) paid on federal grants for the period 2000–2012. We examine the productivity of PhD students who received their degree between 2004 and 2009. Receipt of the PhD is determined by matching the names of PhD students with dissertation records kept in the Caltech library. The name of the advisor is also taken from the dissertation record, as is the discipline of the thesis. We determine gender by matching the first name and approximate date of birth to publicly available Social Security Administration data to determine the probable gender; in cases where we cannot determine gender using this method, we do so by web searches. We observe 933 PhD students enrolled in programs in engineering and the natural sciences who graduated during the interval 2004 to 2009 (Table A in S1 File). Approximately two-thirds receive degrees in engineering or the physical sciences; 29.2% (272 PhD students) are female (Table B in S1 File). Publication data are derived by matching the name of the advisor to Web of Science (WOS). [33]

We attribute to the students articles co-authored with their advisor. In order to provide sufficient time for research to be refereed and published, we measure productivity in year t (pub_t_) as the sum of articles published in years t, t+1 and t+2 divided by 3; the results are robust to different measures (Table L in S1 File). Most students (88.2% percent) publish either while a student or soon after graduating. The average number of publications per year is nearly one article (0.86). We use the 5-year Impact Factor, taken from *Journal Citation Report*, as a measure of quality[33].

We construct a measure of the team with which the focal student works at each year t to explore the role that team composition plays in publication activity. The construct bases team membership on the number of postdoctoral researchers, PhD students, technicians and staff scientists supported on a PI’s federal grants as well as the number of PhD students the PI supervises. (Details provided in S1 File). Team size in year t is then determined by averaging team size for the three preceding years. Our measure of team size excludes the focal student and the advisor. The average size of the team is 8.2, 6.1 of whom are PhD students, 1.9 are postdocs and 0.2 are staff scientists. We find that all but 4 percent of the students who received a PhD during the study period belong to a team. For the sake of simplicity, and with virtually no impact on the results, we restrict the analysis to students with a team. We thus obtain a pooled panel database of 5151 observations for 933 PhD students during their average 5.5 years of PhD study to model their publications during these years.

The dependent variable in the regression analysis is log(1+pub_t_). We use Ordinary Least Squares (OLS) with clustered robust standard errors. The results are virtually unchanged when we rely on Poisson estimation instead of OLS (Table M S1 File). In all regressions we control for discipline, number of years since starting the PhD, year of defense, whether the student had at least one publication in their first year of study, the log of publications of the advisor lagged one year and whether the advisor had any publications in the last three years.

## Results

### Gender of Student

**Fig 1:**
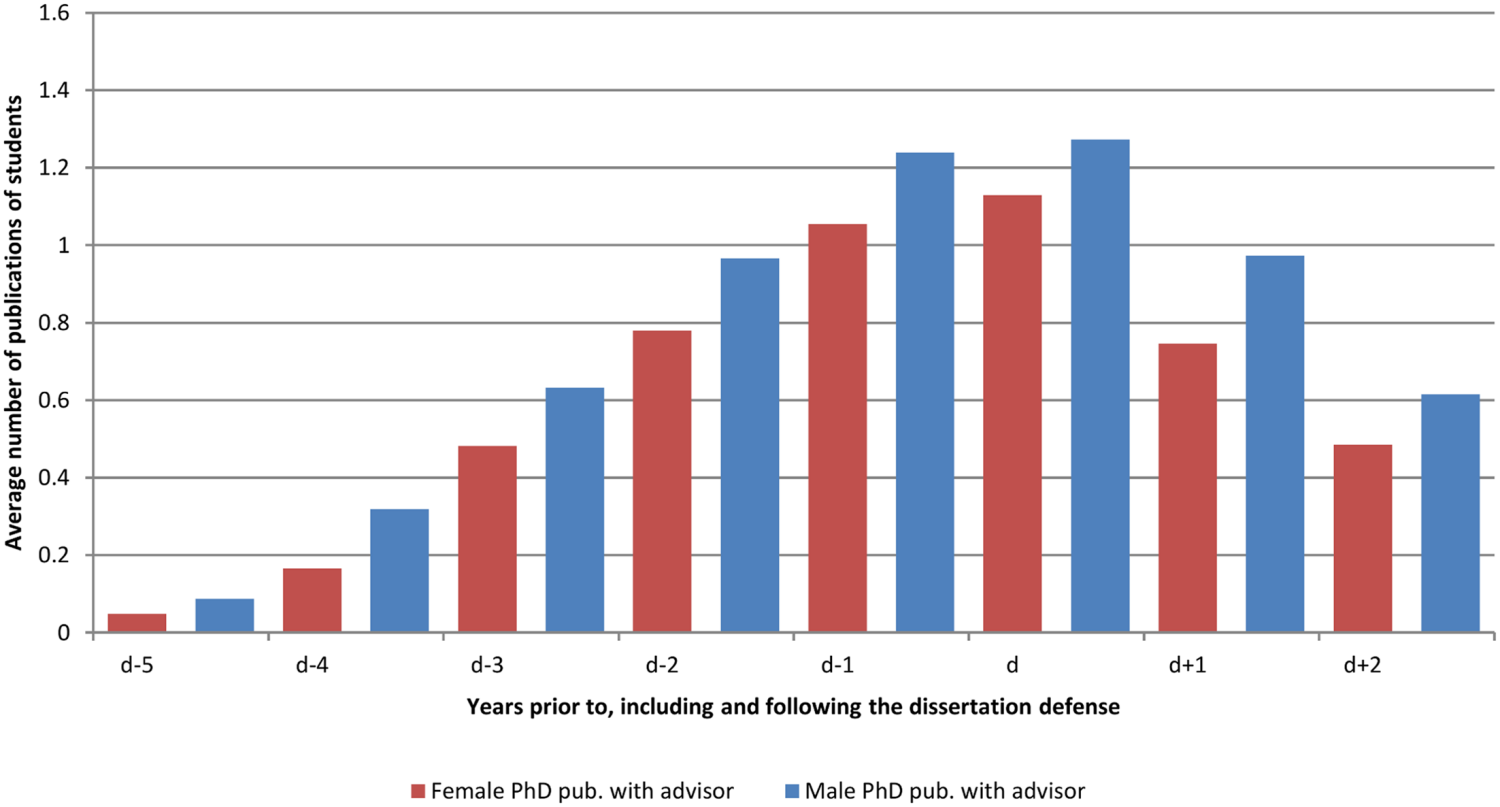
Average number of publications of students classified by gender. The average number of publication is calculated every year, starting from 5 years before the thesis defense (d-5) until two years after the thesis defense (d+2).

**Fig 2:**
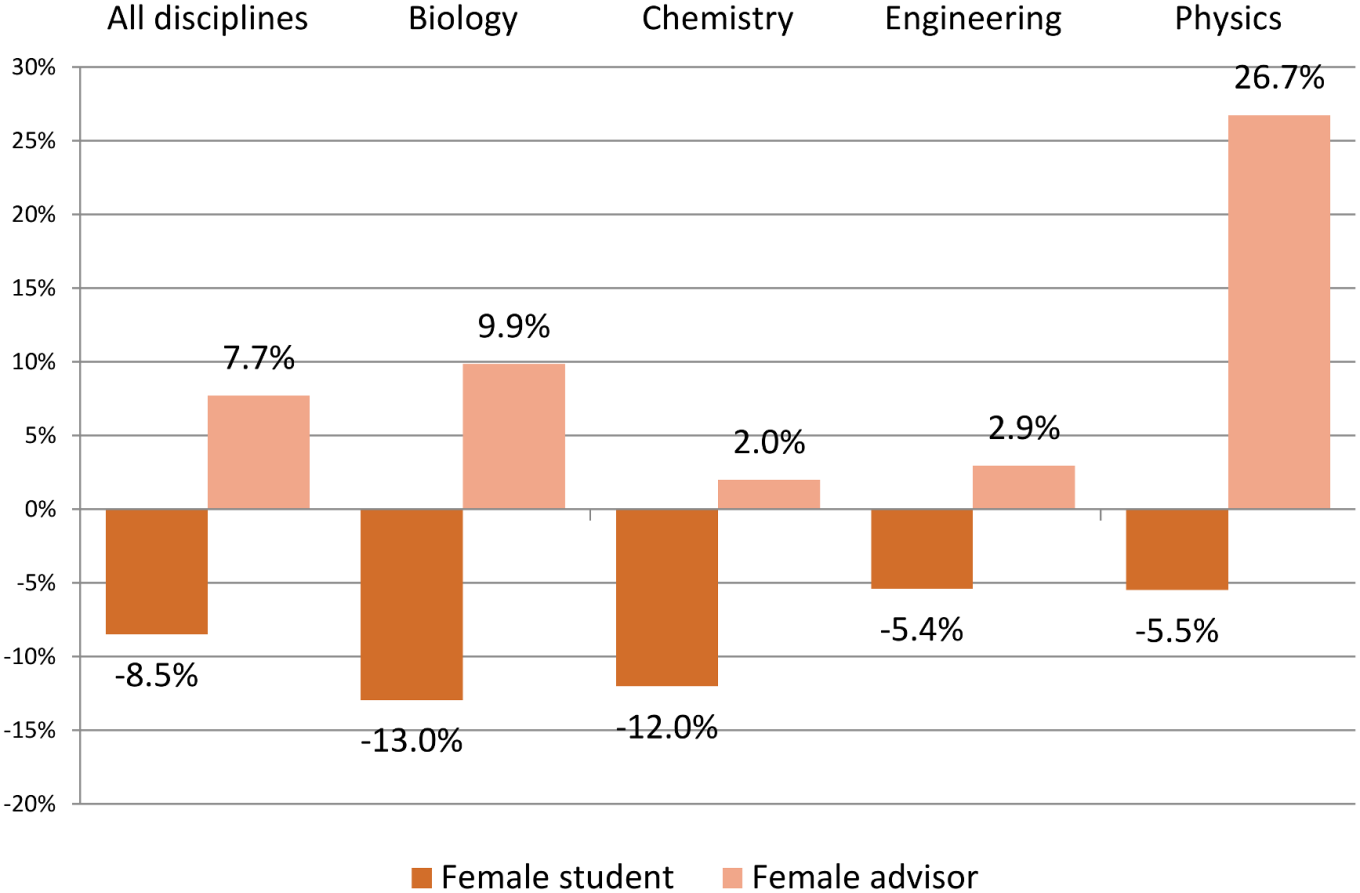
Discipline difference in student publications by gender of student and gender of advisor (Baseline is male student and male advisor). The productivity differences for *all disciplines* are the graphical representation of the marginal effects of the OLS estimation in Table 1, columns 1 and 2. The productivity differences at discipline level are the marginal effect of an OLS estimation where we interact the student and advisor gender dummies with the discipline dummy. Control for advisor productivity, number of years since starting the Ph.D., and year of Ph.D. defense are applied.

In any given year of study, female students publish approximately 8.5% fewer articles than male students (Table 1, column 1). The gender differences exist throughout the graduate career (Fig 1). There are substantial field specific differences: the gender difference is greatest in biology, with a gap of 13%, and least in physics, with a gap of 5.5% (Fig 2). Two other findings from the regression analysis are of particular interest. First, publishing during the first year of a student’s PhD studies is predictive of the student’s publications in his or her subsequent graduate career. Second, students who work with a highly productive advisor publish more than those who work with a less productive advisor.

**Table 1:** Regression Results for Ph.D. productivity. Study sample includes the 933 PhD students who defended their thesis in the period 2004–2009. Control for advisor productivity, discipline, number of years since starting the Ph.D., and year of Ph.D. defense apply.

	(1)	(2)	(3)
VARIABLES	log(1+(t+t1+t2)/3)	log(1+(t+t1+t2)/3)	log(1+(t+t1+t2)/3)
Female student	-0.085***		
Female advisor		0.077**	
M-student M-advisor			*ref*
F-student M-advisor			-0.085***
M-student F-advisor			0.10**
F-student F-advisor			-0.021
More than one advisor	0.020	0.020	0.027
At least one co-advisor	-0.069	-0.072	-0.067
At least one publication first year	0.17***	0.17***	0.17***
log(advisor's publications) (lagged centered)	0.094***	0.094***	0.094***
No advisor pub. in t-1, t-2, t-3	-0.22***	-0.25***	-0.23***
Constant	0.29***	0.27***	0.29***
Observations	5,151	5,151	5,151
R-squared	0.164	0.159	0.167
Dummy discipline	Yes	yes	yes
Number of years since starting PhD	Yes	yes	yes
Year of PhD defense	Yes	yes	yes

** significant at the 5% level;

*** significant at the 1% level

### Gender of Advisor

We observe 204 unique advisors who have supervised one or more dissertations during the period; 12.3% of whom are female. Advisors are highly productive (Table C in S1 File). Male advisors author on average 7.6 articles per year; female advisors author 6.3 publications per year (p < .05). A larger proportion of female advisors (p < .05) have not published in the previous three year period than males (5.0% vs. 2.2%), consistent with previous work finding that female scientists are more likely to exhibit non-publishing spells [2,34] than are men.

**Fig 3:**
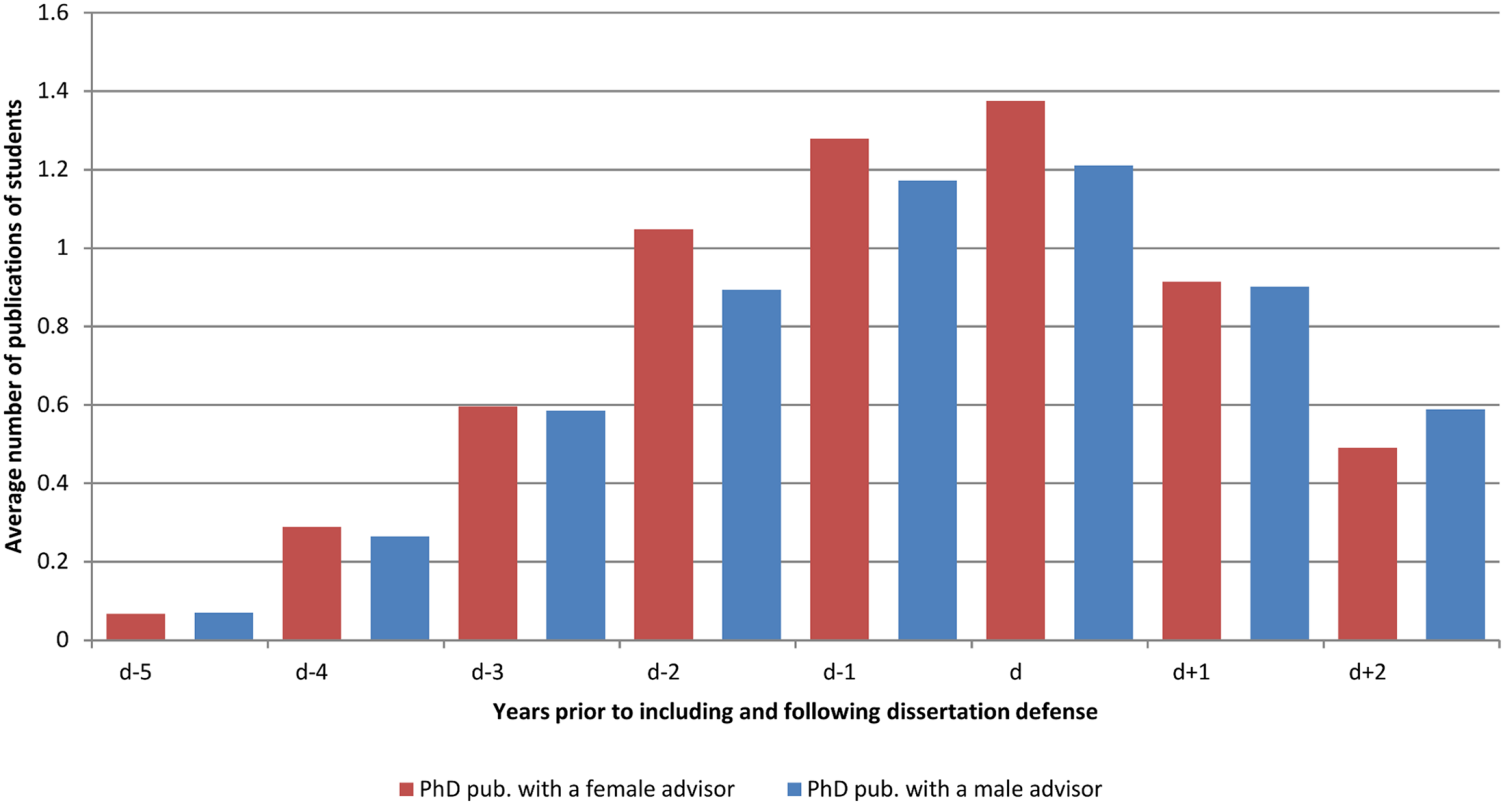
Average number of publications of students classified by gender of advisor. The average number of publication is calculated every year starting from 5 years before the thesis defense (d-5) until two years after the thesis defense (d+2).

The majority of students work with a unique advisor; a small percentage have co-advisors. Eleven percent of the students work with a female advisor. Students working with female advisors publish the same or more than men up to the year they defend their dissertation (Fig 3). As seen in Fig 3, and column 2 of Table 1, we find that students working with a female advisor publish 7.7% more articles a year than do those writing with a male advisor. We find no significant relationship between number of coauthors and the gender of advisor, controlling for characteristics such as field and previous publication history of the advisor and thus reject the hypothesis that the premium is driven by the possible proclivity of women advisors to be more inclusive in defining coauthors. The difference in the publishing productivity of students with female advisors vs. male advisors is greatest in physics and the biological sciences; smallest in engineering and chemistry (Fig 2).

### Gender Dyads

**Fig 4:**
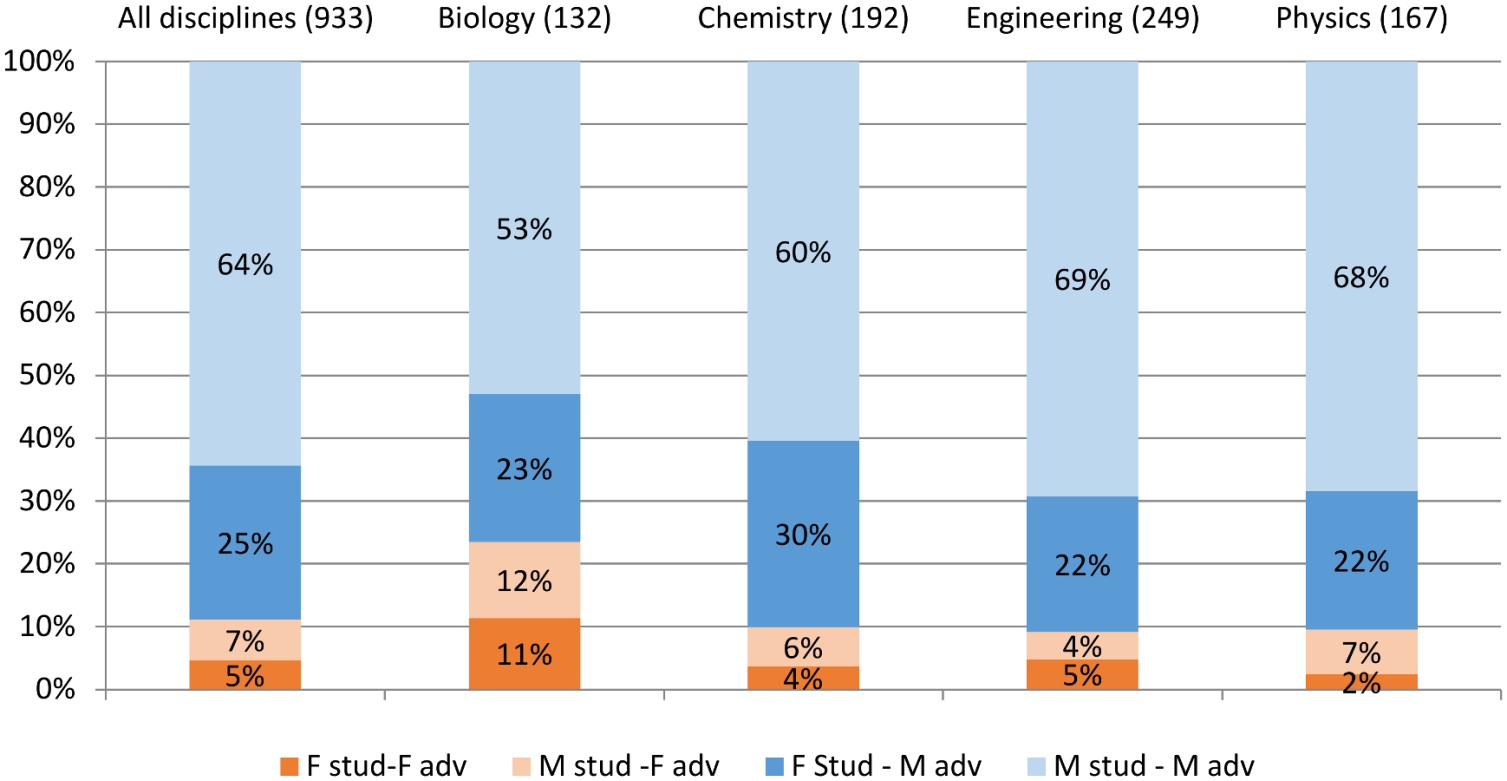
Shares of female/male PhD students with female/male advisors. The shares are calculated for all discipline as well as split by discipline.

We divide students and advisors into four dyads: MM; FF; MF; FM, where the first letter refers to the gender of the student and the second the gender of the advisor. The majority of male students write with a male advisor as do the majority of female students (Fig 4 and Table F in S1 File). The percent of male students working with a female advisor is greatest in biology, smallest in engineering. The percent of female students working with a male advisor is greatest in chemistry and smallest in engineering and physics.

**Fig 5:**
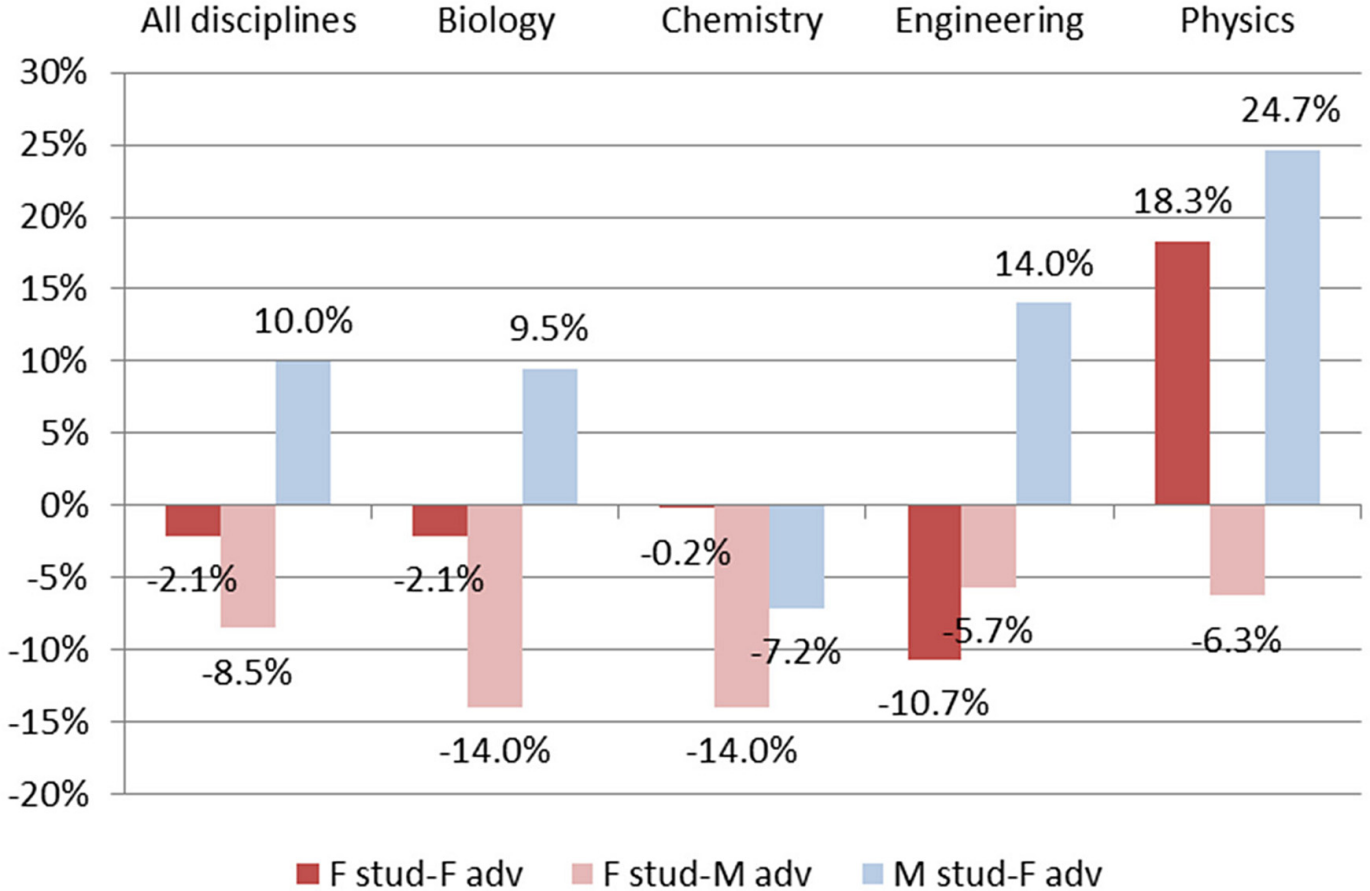
Student publication productivity differences by discipline and by student-advisor gender dyad (baseline male student-male advisor dyad). The productivity differences for *all disciplines* are the graphical representation of the marginal effects of the OLS estimation in Table 1, column 3. The productivity differences at discipline level are the marginal effect of an OLS estimation where we interact the student-advisor gender dyad dummies with the discipline dummy. Control for advisor’s productivity, number of years since starting the Ph.D., and year of Ph.D. defense apply.

For purposes of statistical analysis, we benchmark the FF, FM, MF groups against the MM group. We find that women writing with male advisors publish 8.5% less than males writing with male advisors (p < .01); men writing with female advisors publish 10% more (p < .05); no significant difference exists between women writing with women and the male-male benchmark (Fig 5 and column 3 of Table 1). The female student-male advisor “penalty” is greatest in biology and chemistry; smallest in engineering. The male student-female advisor “premium” is greatest in physics; next greatest in engineering. It is not present, and indeed is negative, in chemistry.

The results indicate that the gender gap is greatest between women writing with male advisors and men writing with female advisors. We are not able to determine if the observed difference is related to the mentoring relationship between the male advisor and the female student, the extent to which the advisor utilizes the expertise of the student or if it is a result of some type of matching whereby women are either assigned or drawn to less productive male advisors or less productive female advisors choose women. Likewise, we are unable to determine whether the premium observed for men writing with female advisors is driven by selection or by female advisors devoting more resources and social capital to male students than they do to female students.

In order to examine if the findings apply to the quality of publications as well as the quantity, we estimate an equation in which the dependent variable is the average 5-year Impact Factor of journals in which the focal student publishes (Table G in S1 File). The results suggest that gender disparity as measured by quality of publications between female students with a male advisor and male students with a female advisor is greater than is the quantity disparity.

### Gender of Team

The average team of the focal PhD student consists of about 8 individuals, approximately three-fourths of whom are PhD students; 22% are postdoctoral researchers. A small percent are staff scientists and technicians. The largest team has almost 35 members, the smallest has one member, excluding the focal PhD student and advisor, who are not counted in measuring team size or gender composition of the team. Approximately 75% of the members of the team are male. The vast majority of teams—83.3%—are mixed in terms of gender. Female advisors are significantly more likely to have a higher proportion of female members on their team– 37.6% versus 28.6% for male advisors; this is consistent with work that shows “homophily” in the formation of authorship patterns [35].

We find no evidence that the publication levels of the student relates to the gender composition of the team (Table K in S1 File). This finding is consistent with the literature suggesting that female dominated teams bring both pluses and minuses to the table in affecting productivity. Moreover, we find no evidence that gender plays a mediating role in terms of the gender composition: Female students neither benefit nor are penalized by working on teams that are increasingly female.

### Discussion

We use new data at the project level to examine the under-researched question of how gender—that of the individual, the advisor and the team—relate to the research productivity of PhD students. We find the direct relationship between gender and publications to be relatively small: women PhD students write approximately 8.5% fewer papers than their male counterparts during their doctoral studies. This is approximately 45 percent less than the gender differential that has recently been reported among faculty [1]. Although the differential is modest, past research on processes of positive feedback and cumulative advantage suggest that the difference will grow over the careers of this recent cohort, not shrink. We also find gender differences to be mediated by the gender of the advisor. Students with female advisors publish more; the premium is only realized by male students, not female students. Moreover, women writing with male advisors publish less than men writing with male advisors. Together these results indicate that the gender gap is greatest between female students working with a male advisor and male students working with a female advisor. The result persists, and indeed is magnified, when we look at the quality of publications, as measured by average Impact Factor, rather than the quantity of publications.

Our data allow us to explore whether the student’s productivity relates to the gender composition of the team with which the student works. Our research is novel in this sense: we are the first to measure team size and gender composition using administrative records. We find no evidence of a significant relationship between the two, where the gender composition of the team is measured in terms of the percent of the team that is female. Moreover, we find no indication that the effect of the gender composition of the team on productivity is mediated by the gender of the student.

Several caveats accompany our research. First, our results are for a highly selective research intensive institution and are not necessarily generalizable to other institutions. Second, we are not able to determine causality. For example, the finding that the gender dyad of the advisor and student plays a role could stem from a variety of causes, such as matching, undervaluation of the research skills of women by male advisors, the amount of energy and effort faculty invest in students or a tendency of women to apply to work under less productive male advisors. They could also arise by gender bias in response to student applications [36]. Third, our measure of team size of the student is biased downward since it does not include postdoctoral researchers who are members of the team but supported on fellowships or other sources of funds rather than grants. Fourth, we only attribute publications to a student if they are coauthored with the dissertation advisor; sole-authored papers or the small number of papers co-authored with other students or faculty who are not the advisors are excluded. Finally, we only use one measure of research productivity—publications. Richer measures, such as job placements and career trajectories are currently being developed.

Our results regarding gender pairing between advisor and advisee bear further research as to why a premium exists for males writing with females and a penalty exists for women writing with men. While we cannot determine causality, the finding raises a cautionary flag to advisors, students and administrators alike and is consistent with recent research showing that gender plays a role in the way in which faculty evaluate students [22] and respond to student inquiries [36]. We hope that other researchers will take up the issue of gender differences among doctoral students, as UMETRICS data, which include an increasing number of research institutions and links to Census data, become available to the research community through the new Institute for Research on Innovation and Science and through Census Research Data Centers.

## Supporting Information

S1 File Table A in S1 File: Distribution of the study sample by year of thesis defense
Table B in S1 File: Distribution of Ph.D. students and advisors by the discipline of the thesis
Table C in S1 File: Average publication productivity of advisor smoothed over 3 years by discipline
Table D in S1 File: Alternative measures of publication productivity of the focal PhD student
Table E in S1 File: Publication productivity and average 5-year Impact Factor (IF) smoothed over 3 years by discipline
Table F in S1 File: Panel of five tables providing information on study sample of PhD students by gender of the student and gender of the advisor
Table G in S1 File: OLS robustness check of gender effects when output is measured as average Impact Factor (avg_IF) of journals in which focal student publishes.
Figure A in S1 File: Team definition
Table H in S1 File: Average share of female team members
Table I in S1 File: Average share of female team members by discipline
Table J in S1 File: Average number of PhD, Postdoc, and staff scientist within the team by gender
Table K in S1 File: Regression Results for Ph.D. productivity, focus on team characteristics
Table L in S1 File: Robustness check of the econometric exercise; alternative measures of dependent variable.
Table M in S1 File: Robustness check of the econometric exercise using Poisson estimates
Table N in S1 File: OLS robustness check of control for advisor “quality”
(DOCX)
