# Supplementary material for: Gender and the Publication Output of Graduate Students: A Case Study
Source: PLoS One. 2016 Jan 13;11(1):e0145146. doi: 10.1371/journal.pone.0145146 (PMC4711938; doi:10.1371/journal.pone.0145146)
Supplement: S1 File — Table A in S1 File: Distribution of the study sample by year of thesis defense Table B in S1 File: Distribution of Ph.D. students and advisors by the discipline of the thesis Table C in S1 File: Average publication productivity of advisor smoothed over 3 years by discipline Table D in S1 File: Alternative measures of publication productivity of the focal PhD student Table E in S1 File: Publication productivity and average 5-year Impact Factor (IF) smoothed over 3 years by discipline Table F in S1 File: Panel of five tables providing information on study sample of PhD students by gender of the student and gender of the advisor Table G in S1 File: OLS robustness check of gender effects when output is measured as average Impact Factor (avg_IF) of journals in which focal student publishes. Figure A in S1 File: Team definition Table H in S1 File: Average share of female team members Table I in S1 File: Average share of female team members by discipline Table J in S1 File: Average number of PhD, Postdoc, and staff scientist within the team by gender Table K in S1 File: Regression Results for Ph.D. productivity, focus on team characteristics Table L in S1 File: Robustness check of the econometric exercise; alternative measures of dependent variable. Table M in S1 File: Robustness check of the econometric exercise using Poisson estimates Table N in S1 File: OLS robustness check of control for advisor “quality” (DOCX) [file pone.0145146.s001.docx]

**Supporting Information**

**Core data**

Our work utilizes three main sources of data from the California Institute of Technology (Caltech): (1) new administrative data that provide detail on student advisors as well as the structure and interactions of their project teams (UMETRICS data), (2) data on publications drawn from a name match to Web of Science and (3) the catalog of dissertations available in the Caltech library.

**UMETRICS**

The new source of administrative data is enhanced STAR METRICS data, or UMETRICS data(32). Only federal funding is covered in the data. The Catalog of Federal Domestic Assistance (CFDA), which is included in each award identifier, provides a full listing of all Federal programs available to universities (and other types of organizations) and is captured in the UMETRICS data to enable filtering federal award expenditures by federal funding agency. Data on the full team of researchers supported on each research grant are captured in the STAR METRICS Level I research institution data (32). This coverage is possible because the data are drawn directly from payroll records. The data also permit the capture of much more detailed information on time allocation and on the interactions of all staff on projects. The STAR METRICS data used here are the file of payroll transactions, which include the occupational classifications of the payees.

The challenges associated with classifying occupations are well known in survey research ), but were carefully coded by the Caltech, and mapped to a standardized set of the following occupational categories: Faculty, Postdoctoral Researcher, Graduate Student, Undergraduate, Staff and Other(32). Gender for staff members and postdoctoral researchers was provided by the Caltech, under carefully controlled conditions with strict access protocols. Gender of faculty was determined from web pages. Gender of students was determined by a probabilistic match of the first name and approximate date of birth to Social Security Administration data to predict gender; in cases where we cannot determine gender using this method, we do so by web searches.

**WEB OF SCIENCE**

The UMETRICS data were matched to ISI Web of Science data, which has information on publications for faculty and graduate students (33). The match criteria included the advisor and student surname, the first letter of the given name and affiliation to Caltech. In order to assure the quality of the match, we hand checked cases of high publication productivity.

**Dissertations**

The UMETRICS data were matched to the Caltech library’s dissertation database, which has information on the student’s advisor and the year the degree was awarded, as well as the classification of the field of research.

**Summary Statistics**

**Advisor and student**

The data consist of 933 individual graduate students who defended their thesis in the period 2004-2009. On average, there are 156 PhD thesis defenses per year. There is an average of 97 distinct advisors per year; the summary statistics by year and field are provided in Tables A and B.

Table A: Distribution of the study sample by year of thesis defense. The number of unique advisors of the theses defended is reported in parenthesis.

| Cohort of defense | PhDs (advisors) |
| --- | --- |
| 2004 | 137 (86) |
| 2005 | 159 (89) |
| 2006 | 163 (100) |
| 2007 | 171 (109) |
| 2008 | 150 (95) |
| 2009 | 153 (96) |
| Total | 933 (204) |

Table B: Distribution of Ph.D. students and advisors by the discipline of the thesis.

| Discipline of the thesis | PhDs | Female PhDs | Advisors | Female Advisors |
| --- | --- | --- | --- | --- |
| BIOLOGY | 132 | 46 (34.8%) | 49 | 9 (18.4%) |
| CHEMISTRY | 192 | 64 (33.3%) | 35 | 5 (14.3%) |
| ENGINEERING | 249 | 66 (26.3%) | 68 | 10 (14.3%) |
| GEOLOGY | 33 | 17 (51.5%) | 26 | 4 (15.4%) |
| MATH | 91 | 17 (18.5%) | 39 | 1 (2.5%) |
| OTHERS | 69 | 21 (31.9%) | 25 | 4 (16%) |
| PHYSICS | 167 | 41 (24.4%) | 62 | 6 (9.5%) |
| *TOTAL* | *933* | *272 (29.1%)* |  |  |

Advisors in Table B sum to 304 PIs. Some of the PIs are double-counted because one PI can have students with theses classified in different subjects. For instance, the same PI might have two students, one with the thesis classified in the field of biology and one with the thesis classified in the field of engineering.

As is evident from a perusal of Table B, a large part of the students’ theses is classified in the field of physics, engineering, chemistry and biology. We find that the majority of students (92%) work with a unique advisor; 4% of the students have a co-advisor.

**Advisor publication productivity**

Table C shows the advisors’ average publication productivity smoothed over 3 years. This variable is included as a proxy of the advisor’s productivity in the regression exercises. Male advisors are more productive than female advisors (7.63 articles per year VS 6.32 articles per year; p<.05).

Table C: Average publication productivity of the advisor smoothed over 3 years by discipline. The percentage of the non-publishing time spells (i.e. periods when the advisor does not publish) is reported.

| Gender of advisor (N. of advisors) | Average publications per year in | % of non-publishing time spells |
| --- | --- | --- |
| Total | t-1, t-2, t-3 | t-1, t-2, t-3 |
| F (25) | 6.32 | 5.00% |
| M (179) | 7.63 | 2.20% |
| Biology |  |  |
| F (9) | 6.63 | 11% |
| M (40) | 8.53 | 0% |
| Chemistry |  |  |
| F (5) | 10.2 | 0% |
| M (30) | 12.09 | 0% |
| Engineering |  |  |
| F (10) | 5.66 | 0% |
| M (58) | 7.73 | 0% |
| Physics |  |  |
| F (6) | 5.47 | 0% |
| M (56) | 7.53 | 0% |

**Student - Advisor publication productivity**

We attribute the publications of PhD advisors to their students if the student’s name appears among the co-authors. This attribution strategy reduces possible homonymy problems in the publication attribution.

Table D shows average publication productivity of PhD students according to three alternative measures: count of publications in year t, count of publication per year in t+1, t+2 and count of publications per year in t, t+1, t+2. Our study sample is a panel of 5151 observations corresponding to a dyad of PI- PhD student*years.

Table D: Alternative measures of publication productivity of the focal PhD student

| Variable | Obs | Mean | Std. Dev. | Min | Max |
| --- | --- | --- | --- | --- | --- |
| *Publication productivity smoothed over 3 years* |  |  |  |  |  |
| Publications (t+t1+t2)/3 | 5151 | 0.86 | 1.08 | 0.00 | 11.67 |
| Dummy publications>0 in t or (t+1) or (t+2) | 5151 | 0.71 | 0.45 | 0.00 | 1.00 |
| *Publication productivity in t* |  |  |  |  |  |
| Publications t | 5151 | 0.77 | 1.35 | 0.00 | 18.00 |
| *Publication productivity smoothed over 2 years* |  |  |  |  |  |
| Dummy publications>0 in t | 5151 | 0.39 | 0.49 | 0.00 | 1.00 |
| Publications (t1+t2)/2 | 5151 | 0.91 | 1.19 | 0.00 | 13.00 |
| Dummy publications>0 in (t+1) or (t+2) | 5151 | 0.64 | 0.48 | 0.00 | 1.00 |

We select average publications per year in t, t+1, t+2 as the dependent variable in our econometric models. Table E shows publication productivity and average Impact Factor according to thesis subject.

Table E: Publication productivity and average 5-year Impact actor (IF) smoothed over 3 years by discipline

| Dependent variable | Obs | Mean | Std. Dev. | Min | Max |
| --- | --- | --- | --- | --- | --- |
| Publications (t+t1+t2)/3 | 5151 | 0.86 | 1.08 | 0 | 11.67 |
| Dummy publications>0 in t or (t+1) or (t+2) | 5151 | 0.71 | 0.46 | 0 | 1 |
| Average IF (t+t1+t2)/3 | 5151 | 2.26 | 3.00 | 0 | 26.18 |
| Dummy average IF>0 in t or (t+1) or (t+2) | 5151 | 0.64 | 0.48 | 0 | 1 |
| **Biology** |  |  |  |  |  |
| Publications (t+t1+t2)/3 | 706 | 0.65 | 0.64 | 0 | 3.33 |
| Dummy publications>0 in t or (t+1) or (t+2) | 706 | 0.72 | 0.45 | 0 | 1 |
| Average IF (t+t1+t2)/3 | 706 | 3.07 | 3.81 | 0 | 25.5 |
| Dummy average IF>0 in t or (t+1) or (t+2) | 706 | 0.67 | 0.47 | 0 | 1 |
| **Chemistry** |  |  |  |  |  |
| Publications (t+t1+t2)/3 | 1068 | 1.00 | 0.93 | 0 | 6.67 |
| Dummy publications>0 in t or (t+1) or (t+2) | 1068 | 0.83 | 0.37 | 0 | 1 |
| Average IF (t+t1+t2)/3 | 1068 | 3.58 | 3.08 | 0 | 22.34 |
| Dummy average IF>0 in t or (t+1) or (t+2) | 1068 | 0.82 | 0.38 | 0 | 1 |
| **Engineering** |  |  |  |  |  |
| Publications (t+t1+t2)/3 | 1358 | 0.91 | 1.16 | 0 | 8.67 |
| Dummy publications>0 in t or (t+1) or (t+2) | 1358 | 0.70 | 0.46 | 0 | 1 |
| Average IF (t+t1+t2)/3 | 1358 | 1.28 | 1.99 | 0 | 15.59 |
| Dummy average IF>0 in t or (t+1) or (t+2) | 1358 | 0.56 | 0.49 | 0 | 1 |
| **Physics** |  |  |  |  |  |
| Publications (t+t1+t2)/3 | 959 | 1.04 | 1.47 | 0 | 11.67 |
| Dummy publications>0 in t or (t+1) or (t+2) | 959 | 0.70 | 0.46 | 0 | 1 |
| Average IF (t+t1+t2)/3 | 959 | 2.58 | 3.30 | 0 | 26.18 |
| Dummy average IF>0 in t or (t+1) or (t+2) | 959 | 0.67 | 0.47 | 0 | 1 |

Table F provides summary information about the nature of the PhD student – advisor gender dyad. The first panel, Table F, shows the distribution by gender dyads. The most frequent class is male students supervised by male advisors (64%). Figure 3 in the paper shows the same distribution according to the different disciplines. The second panel, Table F, shows the average publication productivity according to Publications (t+t1+t2)/3. The third panel, Table F, shows the percentage of observations PhD student*year where the average publication productivity (Publications (t+t1+t2)/3) is larger than zero. The dyad female advisors supervising male PhD students shows the highest percentage of publication time spells; 83% of the observations the publication productivity of this class are larger than zero. The fourth panel, Table F, shows the percentage of dyads PhD student – advisor gender for which the PhD student has at least one publication in her first year of PhD. Female advisors supervise a higher percentage of students with at least one publication during the first PhD year. The fifth panel, Table F, shows the average impact factor by gender.

Table F Panel of five tables providing information on the study sample of PhD students by gender of the student and gender of the advisor. 1) shows the sample distribution according to the gender dyads. 2) shows the student’s average publication productivity in year t. 3) shows PhD student’s percentage of publication time spells (i.e. percentage of observations where publication productivity is > 0). 4) shows the percentage of PhD students who have at least one publication during their first PhD year. 5) shows the average impact factor on which the student publishes in year t.

| PhD student distribution |  |  |  |  |  | PhD students' productivity | |  |  |  |
| --- | --- | --- | --- | --- | --- | --- | --- | --- | --- | --- |
| 1) |  | gender Advisor | |  |  | 2) |  | Gender advisor | |  |
|  |  | F | M | total |  |  |  | F | M | Total |
| gender PhD | F | 43(5%) | 229(25%) | 272 |  | gender PhD | F | 0.74 | 0.74 | 0.74 |
|  | M | 61(7%) | 600(64%) | 661 |  |  | M | 1.07 | 0.89 | 0.91 |
|  | total | 104 | 829 | 933 |  |  | total | 0.93 | 0.85 | 0.86 |
|  |  |  |  |  |  |  |  |  |  |  |
| % PhD students’ publication time spells | | | |  |  | % PhD students with at least one publication in the first year of PhD | | | | |
| 3) |  | gender advisor | |  |  | 4) |  | gender advisor | |  |
|  |  | F | M | total |  |  |  | F | M | total |
| gender PhD | F | 70.10% | 66.80% | 67.30% |  | gender PhD | F | 44.20% | 38.80% | 39.70% |
|  | M | 83.20% | 71.30% | 72.40% |  |  | M | 45.90% | 35.60% | 36.60% |
|  | total | 77.90% | 70.10% | 70.90% |  |  | total | 45.20% | 36.50% | 37.50% |
|  |  |  |  |  |  |  |  |  |  |  |
| Average Impact factor |  |  |  |  |  |  |  |  |  |  |
| 5) |  | Gender advisor | |  |  |  |  |  |  |  |
|  |  | F | M | total |  |  |  |  |  |  |
| gender PhD | F | 2.48 | 2.07 | 2.13 |  |  |  |  |  |  |
|  | M | 3.5 | 2.18 | 2.31 |  |  |  |  |  |  |
|  | total | 3.09 | 2.15 | 2.26 |  |  |  |  |  |  |

**Econometric Exercises Using Student and Advisor Variables**

Table 1 in the text shows the results of three regressions relating the effect of the gender of the student and gender of the supervisor on the student’s publication productivity; the dependent variable considered is log(1+Publications (t+t1+t2)/3). Robust standard errors are calculated. Column 1 examines the impact of the gender of the student on publication productivity controlling for student and advisor characteristics, column 2 examines the impact of the gender of the advisor on publication productivity controlling for student and advisor characteristics and column 3 examines the impact of the gender dyad of student and advisor controlling for student and advisor characteristics.

In order to examine if the gender differential exists for quality as well as for publication, we estimate an equation in which the dependent variable is the average 5-year Impact Factor (avg_IF) of journals in which the focal student publishes (Table G). The mean of the variable is 2.26; the variance is 3.00. The lag structure and all independent variables remain the same as those used in Table 1 in the text. The results are presented in Table G. In each of the three equations the signs of the three gender variables remain the same as those in Table 1; the level of significance of the female advisor variable and its interaction with a male student increases as well as the absolute size of the female advisor coefficient and the male student, female-advisor-coefficient which more than double. The results suggest that gender disparity as measured by quality of publications between female students with a male advisor and male students with a female advisor is substantially greater than is the quantity disparity.

Table G OLS robustness check of gender effects when output is measured as average impact factor (avg_IF) of journals in which focal student publishes.

| avg_IF | (1) | (2) | (3) |
| --- | --- | --- | --- |
|  | log(1+IF(t+t1+t2)/3) | log(1+IF(t+t1+t2)/3) | log(1+IF(t+t1+t2)/3) |
| Female student | -0.11*** |  |  |
| Female advisor |  | 0.22*** |  |
| M-student M-advisor |  |  | *ref* |
| F-student M-advisor |  |  | -0.11*** |
| M-student F-advisor |  |  | 0.27*** |
| F-student F-advisor |  |  | 0.083 |
| More than one advisor | -0.033 | -0.024 | -0.015 |
| At least one co-advisor | -0.15* | -0.15** | -0.14* |
| At least one publication First year | 0.19*** | 0.18*** | 0.18*** |
| log(advisor's publications) (lagged centered) | 0.19*** | 0.19*** | 0.19*** |
| No advisor pub. in t-1, t-2, t-3 | -0.48*** | -0.55*** | -0.52*** |
| Constant | 0.28*** | 0.24*** | 0.26*** |
| Observations | 5,151 | 5,151 | 5,151 |
| R-squared | 0.254 | 0.257 | 0.262 |
| Dummy discipline | yes | yes | yes |
| Number of years since starting PhD | yes | yes | yes |
| Year of PhD defense | yes | yes | yes |

**Team**

We exploit the rich grant information to characterize the team associated with a focal student as well as information taken from library records concerning theses that faculty advised. We assign to the PhD in year t the team of her advisor, including all the employees paid and/or supervised by the faculty member. When an employee is paid by two or more faculty we assign her to the advisor, or to the faculty who is paying her more (about 10% of the cases). For the students who are supervised by the advisor but not paid on public grants by the advisor, we assume that they are part of the team in the five years before the year of the thesis defence (d).

After attributing a team to a focal PhD student for each year, we smooth the variables measuring the average team composition over 3 years: t-1, t-2, t-3.

The following example (Figure A) elucidates the team definition applied for a focal student (PhD 5) in year t. The green circle (PhD 5) is the focal student, supervised by faculty member a, for whom we want to define the team. Red circles are PhDs who have faculty member a as an advisor, blue circles are PhD students paid on public grants but who have yet to defend (we do not have information about their thesis, therefore we do not know who their advisor is). Blue lines represent payments to employees on public grants. In this case, and assuming that PhD student 4 is paid more on grants from faculty a than grants from faculty b, the size of focal PhD 5’s team is 6.

Fig A: Graphical representation of team definition. In the current example the focal student is the student labelled PhD 5 (green circle). Red circles represent students who are supervised by Faculty a. Blue lines represent payments with public grant funds from the Faculty to employees. In year *t* the team of the PhD5’s advisor (Faculty a), includes all the employees paid and/or supervised by Faculty a, i.e. PhD0, PhD1, PhD2, PhD3, PhD4, Staff scientist 1, and Postdoc3. When an employee is paid by two or more Faculty we assign her to the Faculty who is paying her more (about 10% of the cases). For instance, if PhD4 is paid more by Faculty a on Grant c, than by Faculty b on Grant D, then she will be assigned to Faculty a’s team. The team of the Faculty a is assigned to the focal student PhD5.


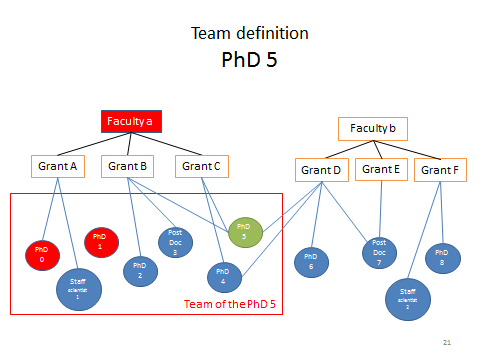


This approach generates a total of 5151 observations. Of these 4289 observations (83.27%) are characterized by mixed teams (male and female members) assigned to the focal PhD student. Table H shows the percentage of female employees in the team for the whole sample (5151 observations, third column) and conditional on observing a mixed team (4289 observations, forth column). Table I shows equivalent statistics to Table H but at the discipline level.Table H: Average share of female team members (*Avg. share of females in the team*) and average share of female team members considering only the teams that include at least one female and one male team member (*Avg. share of females in the mixed teams*)

|  | Observations | Avg. share of females in the team | Avg. share of females in the mixed teams  (4289 0bs.) |
| --- | --- | --- | --- |
| ALL | 5151 | 25.40% | 29.70% |
| Female advisor | 576 | 34.40% | 37.60% |
| Male advisor | 4575 | 24.30% | 28.70% |

Table I: Average share of female team members (*Avg. share of females in the team*) and average share of female team members considering only the teams that include at least one female and one male team member (*Avg. share of females in the mixed teams*) by discipline

|  | observations | Avg. share of females in the team | Avg. share of females in the mixed teams |
| --- | --- | --- | --- |
| **Biology** |  |  |  |
| ALL | 706 | 31% | 34% |
| Female advisor | 166 | 41% | 43% |
| Male advisor | 540 | 28% | 31% |
| **Chemistry** |  |  |  |
| ALL | 1068 | 28% | 29% |
| Female advisor | 107 | 44% | 45% |
| Male advisor | 916 | 26% | 28% |
| **Engineering** |  |  |  |
| ALL | 1358 | 23% | 28% |
| Female advisor | 122 | 30% | 35% |
| Male advisor | 1236 | 23% | 27% |
| **Physics** |  |  |  |
| ALL | 959 | 20% | 27% |
| Female advisor | 95 | 20% | 24% |
| Male advisor | 864 | 20% | 27% |

Table J shows the average number of team members in t-1, t-2, t-3 grouped in 6 different classes: Male / female PhD students, Male / female postdocs, Male / female staff scientists. The average size of all teams is 8.17 employees per year. A large proportion of the employees are PhD students.

Table J: Average number of PhD, Postdoc, and staff scientist within the team by gender

|  | Obs. | Avg. n. of team members | Std. Dev. | Min | Max |
| --- | --- | --- | --- | --- | --- |
| 1) Male PhD | 5151 | 4.41 | 3.68 | 0 | 18.67 |
| 2) Female PhD | 5151 | 1.66 | 1.85 | 0 | 9.33 |
| 3) Male postdoc | 5151 | 1.37 | 1.57 | 0 | 7.67 |
| 4) Female postdoc | 5151 | 0.5 | 0.81 | 0 | 8.67 |
| 5) Male staff scientists | 5151 | 0.19 | 0.53 | 0 | 4.67 |
| 6) Female staff scientists | 5151 | 0.05 | 0.2 | 0 | 2.67 |
| *1+2+3+4+5+6* |  | *8.17* |  |  |  |
|  |  |  |  |  |  |
| *Male* (1+3+5) | 5151 | 5.97 | 4.9 | 0 | 27 |
| *Female* (2+4+6) | 5151 | 2.2 | 2.32 | 0 | 11.67 |
| *Male + Female* |  | *8.17* |  |  |  |
|  |  |  |  |  |  |
| Team size | 5151 | *8.17* | 6.31 | 0.33 | 34.67 |

**Econometric Exercises Using Team Variables**

We include in the regression three variables that characterize the team composition:

- - the team size that consists in the count of team members, excluding focal student and advisor (smoothed over t-1, t-2, t-3)
  - the presence of at least one postdoc in the team (in t-1, t-2, t-3)
  - the logit-share of women in the team (smoothed over t-1, t-2, t-3)

Note that the logit-share of female in the team is equal to log(share/(1-share)) where share is the count of female employees over the total number of employees in the team. The coefficients estimated in the regression model can be interpreted as semi-elasticities according to the following rewriting of the regression equation:

$$\log\left( y \right)=\beta*\log\left( \frac{share}{1-share} \right)+\gamma*controls$$

$$y=e^{\beta*\log\left( \frac{share}{1-share} \right)+\gamma*controls}$$

$$y={(\frac{share}{1-share})}^{\beta}*e^{\gamma*controls}$$

Marginal effects calculation:

$$\frac{dy}{dshare}=\beta*\left( \frac{share}{1-share} \right)^{\beta-1}*\frac{share}{share*\left( 1-share \right)^{2}}*e^{controls}$$

$$\frac{dy}{dshare}=\beta*\left( \frac{share}{1-share} \right)^{\beta}*\frac{1}{share*\left( 1-share \right)}*e^{controls}$$

$$\frac{dy}{dshare}=\beta*y*\frac{1}{share*\left( 1-share \right)}$$

$$\frac{dy/y}{dshare}=\frac{\Delta y\%}{\Delta s}=\beta*\frac{1}{share*\left( 1-share \right)}$$

Note that the logit-share estimated coefficient $\beta$ must be interpreted as a semi-elasticity when multiplied by $\frac{1}{share*\left( 1-share \right)}$. The share value selected for the calculation of the marginal effects is 0.2 (the median value of the share). Table K shows the effects of the logit-share (Column 1) and logit-share interacted with the dyads gender student - gender advisor (Column 2). Neither the coefficient log(Share/(1-Share)) alone, nor the interactions with the dyads student gender – advisor gender are significant. We conclude that we can reject the hypothesis that team gender composition has an impact on the student’s productivity.

Table K: Regression Results for Ph.D. productivity. We include team gender composition variables among the regressors. Team composition is measured as log(share/(1-share)), where share is the count of female over the total number of team members. The study sample includes the 933 PhD students who defended their thesis in the period 2004-2009. Control for advisor quality, discipline, number of years since starting the Ph.D., and year of Ph.D. defense apply.

|  | (1) | (2) |
| --- | --- | --- |
| VARIABLES | log(1+(t+t1+t2)/3) | log(1+(t+t1+t2)/3) |
| M-student M-advisor | Ref. | Ref. |
| F-student M-advisor | -0.086*** | -0.084*** |
| M-student F-advisor | 0.096** | 0.100** |
| F-student F-advisor | -0.024 | -0.019 |
| log(advisor's publications) (lagged centered) | 0.092*** | 0.092*** |
| No advisor pub. in t-1, t-2, t-3 | -0.23*** | -0.22*** |
| More than one advisor | 0.020 | 0.020 |
| At least one co-advisor | -0.068 | -0.070 |
| Never paid on public funds | -0.11*** | -0.11*** |
| At least one publication First year | 0.16*** | 0.16*** |
| log(team size) (lagged centered) | 0.0048 | 0.0050 |
| At least one PostDoc in the team (lagged) | 0.015 | 0.014 |
| log(Share/(1-Share)) (lagged centered) | -0.00037 |  |
| M-student M-advisor * log(share/(1-share)) (lagged centered) |  | 0.00093 |
| M-student F-advisor * log(share/(1-share)) (lagged centered) |  | -0.027 |
| F-student M-advisor * log(share/(1-share)) (lagged centered) |  | 0.0069 |
| F-student F-advisor * log(share/(1-share)) (lagged centered) |  | -0.0081 |
| Constant | 0.31*** | 0.31*** |
| Observations | 5,151 | 5,151 |
| R-squared | 0.173 | 0.174 |
| Dummy discipline | yes | yes |
| Number of years since starting PhD | yes | yes |
| Year of PhD defense | yes | yes |

**Robustness checks**

We run two robustness checks for the model specification that includes the gender dyads by selecting two alternative lag structures of the dependent variable: Average publications in a two year span (t1+t2)/2 and current publications in time t (Table L). The results are qualitatively the same, although the M-student F-advisor dummy is not significant for the short publication window. This may be due to the presence of a high percentage of zeros in the dependent variable (about 60%) when measured in this time frame.

Table L: Robustness check of the econometric exercise. We consider two alternative publication productivity measures: count of publications in two year span (*log(1+(t1+t2)/2)*) and count of publications in the current year (*log(1+Pub. t)*). Control for advisor productivity, discipline, number of years since starting the Ph.D., and year of Ph.D. defense apply.

|  | (1) | (2) |
| --- | --- | --- |
| VARIABLES | log(1+Pub. t) | log(1+(t1+t2)/2) |
| M-student M-advisor | ref | ref |
| F-student M-advisor | -0.16*** | -0.18*** |
| M-student F-advisor | 0.13 | 0.20* |
| F-student F-advisor | -0.021 | -0.091 |
| log(advisor's publications) (lagged centered) | 0.17*** | 0.23*** |
| No advisor pub. in t-1, t-2, t-3 | -0.32*** | -0.38*** |
| More than one advisor | 0.064 | 0.11 |
| At least one co-advisor | -0.16 | -0.17 |
| At least one publication First year | 0.55*** | 0.32*** |
| Constant | 0.12 | 0.63*** |
| Observations | 5,151 | 5,151 |
| R-squared | 0.145 | 0.105 |
| Dummy discipline | yes | yes |
| Number of years since starting PhD | yes | yes |
| Year of PhD defense | yes | yes |

We also run Poisson estimations including the dependent variable in count format, that is the count of the articles published in t, t+1, t+2. The results are reported in the Table M, and are not substantively different.

Table M: Robustness check of the econometric exercise. We consider as productivity measure the count of publication in the three years t, t+1, t+2 and we use Poisson as alternative estimation approach.Control for advisor productivity, discipline, number of years since starting the Ph.D., and year of Ph.D. defense apply.

|  | (1) |
| --- | --- |
|  | count(Pub. t+t1+t2) |
| M-student M-advisor | ref |
| F-student M-advisor | -0.21*** |
| M-student F-advisor | 0.21** |
| F-student F-advisor | -0.081 |
| log(advisor's publications) (lagged centered) | 0.27*** |
| No advisor pub. in t-1, t-2, t-3 | -0.82** |
| More than one advisor | 0.11 |
| At least one co-advisor | -0.25 |
| At least one publication First year | 0.42*** |
| Constant | 0.29** |
| Observations | 5,151 |
| Dummy discipline | yes |
| Number of years since starting PhD | yes |
| Year of PhD defense | yes |

We run an additional robustness check to control for the “quality” of the advisor as measured by experience in supervising dissertations (Table N). The variable “count of supervised theses” is the count of all the PhD theses supervised by the advisor of the focal student previous to year t. A limitation of this variable is that because we retrieved the thesis information from the library archives of Caltech we are not able to trace theses supervised by the advisor prior to joining the institution. We include in the regression a dummy “first student” that equals one when the “count of supervised theses” equals zero, that is when the focal student is the first PhD student supervised by the advisor at this institution; 14.%% of the 933 focal students in the sample are first students. In addition, we include a dummy that equals 1 when the focal student is the first and only student of the advisor in the observation period (6% of all students).

We find the impact of the “count of supervised theses” variable to be positive: a 10% increase in the number of students supervised in the past increases the productivity of the focal student by 0.31%. The dummy variable “first student” shows that the first student of the advisor performs significantly better than the following students. We find no impact of being the only PhD student of the advisor. Of particular importance for this research is that the quality controls are orthogonal to the three gender variables of interest and their inclusion in the regression does not change the substance of our conclusions.

Table N: OLS robustness check of control for advisor “quality” measured by number of students supervised, whether student is first student and whether student is the only student supervised.

|  | (1) | (2) | (3) | (4) |
| --- | --- | --- | --- | --- |
| VARIABLES | log(1+(t+t1+t2)/3) | log(1+(t+t1+t2)/3) | log(1+(t+t1+t2)/3) | log(1+(t+t1+t2)/3) |
| Female student | -0.085*** |  |  |  |
| Female advisor |  | 0.077** |  |  |
| M-student M-advisor |  |  | *ref* | *ref* |
| F-student M-advisor |  |  | -0.085*** | -0.087*** |
| M-student F-advisor |  |  | 0.10** | 0.11** |
| F-student F-advisor |  |  | -0.021 | -0.028 |
| More than one advisor | 0.020 | 0.020 | 0.027 | 0.026 |
| At least one co-advisor | -0.069 | -0.072 | -0.067 | -0.056 |
| At least one publication First year | 0.17*** | 0.17*** | 0.17*** | 0.16*** |
| log(advisor's publications) (lagged centered) | 0.094*** | 0.094*** | 0.094*** | 0.094*** |
| No advisor pub. in t-1, t-2, t-3 | -0.22*** | -0.25*** | -0.23*** | -0.25*** |
| log(count of supervised theses) |  |  |  | 0.031** |
| First student |  |  |  | 0.15*** |
| Only student |  |  |  | -0.061 |
| Constant | 0.29*** | 0.27*** | 0.29*** | 0.25*** |
| Observations | 5,151 | 5,151 | 5,151 | 5,151 |
| R-squared | 0.164 | 0.159 | 0.167 | 0.177 |
| Dummy discipline | yes | yes | yes | yes |
| Number of years since starting PhD | yes | yes | yes | yes |
| Year of PhD defense | yes | yes | yes | yes |
